# Predictive mechanistic modelling of vegetable oil autoxidation using a parametrised kinetic network

**DOI:** 10.1016/j.crfs.2025.101233

**Published:** 2025-10-30

**Authors:** Vincent J.P. Boerkamp, Khoa A. Nguyen, Jean-Paul Vincken, John P.M. van Duynhoven, Marie Hennebelle

**Affiliations:** a Laboratory of Food Chemistry, Wageningen University & Research, Bornse Weilanden 9, Wageningen, 6708 WG, The Netherlands; b Laboratory of Biophysics, Wageningen University & Research, Stippeneng 4, Wageningen, 6708 WE, The Netherlands; c Unilever Food Innovation Centre, Bronland 14, Wageningen, 6708 WH, The Netherlands

**Keywords:** Lipid oxidation, Kinetic modelling, NMR spectroscopy, Triolein, Trilinolein, Trilinolenin, Hydroperoxides, Aldehydes

## Abstract

Developing measures to prevent oxidation of vegetable oils currently requires cumbersome empirical testing of shelf-life. In order to rationalise the design of antioxidative strategies, we developed a predictive model that accounts for environmental (oxygen pressure, headspace to oil ratio, temperature) and compositional (fatty acid composition, antioxidants, metal concentration) parameters. A kinetic network comprising 23 oxidation reactions was constructed and parametrised by estimating the corresponding activation energies of these reactions for triolein, trilinolein, and trilinolenin. The parametrised model accurately predicted the autoxidation reactions of vegetable oils with different compositions and environmental conditions (normalised root mean square errors < 0.05). Such model will enable *in silico* shelf-life predictions of vegetable oils, can be parametrised for other edible oils and ultimately extended to emulsified foods.

## Highlights

• A kinetic network is constructed of major lipid autoxidation reactions.
• The network includes hydroperoxides, aldehydes, epoxides, hydroxides, and ketones.
• Activation energies are provided for triolein, trilinolein, and trilinolenin.
• The model is parametrised for temperature and fatty acid composition.
• The model enables shelf-life prediction of vegetable oils with antioxidants.

## Graphical abstract

**Figure:**
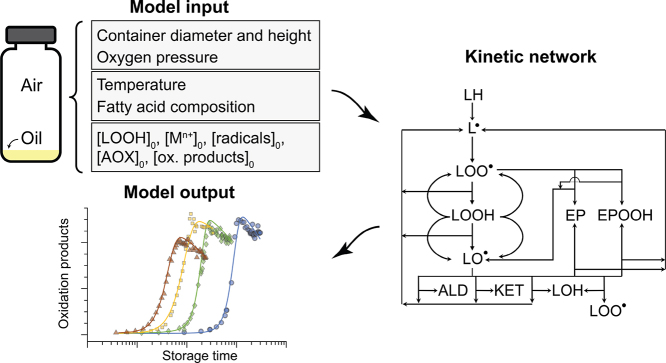

## 1 Introduction

Lipid oxidation is the main cause of vegetable oil degradation in foods (Hennebelle et al., 2024), and derived products such as biolubricants (Hamnas and Unnikrishnan, 2023), and biofuels (Yaakob et al., 2014). Current mitigation strategies such as chilled storage, protective packaging and synthetic antioxidants, are expensive and do not meet the increasing demand of consumers for more sustainable and natural options (Ghelichi et al., 2023). The design of alternative natural antioxidant routes is hampered by a lack of understanding of critical oxidation pathways as well as cumbersome experimental shelf-life testing.

**Fig. 1:**
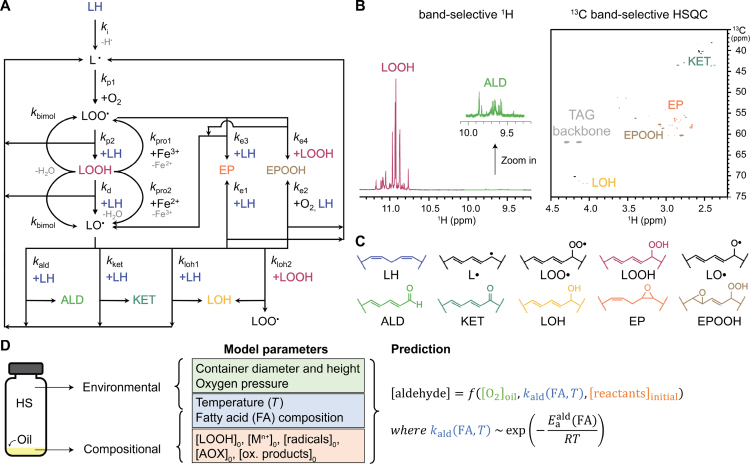
Schematic depiction of the concept of this paper. (A) Kinetic reaction network of lipid autoxidation with kinetic constants ‘k’. Radical recombinations between  , and  that can terminate radical chains were included (Eq. (19), (20), (21)), but for clarity not included in the scheme. All reactions were described as ordinary differential equations (ODEs) using a mixture of first and second order reactions (Eq. (24), (25), (26), (27), (28), (29), (30)). Nguyen et al. (2024b) (B) Two experiments (band-selective 1D ${}^{1}$H NMR, (Merkx et al., 2018) and band-selective ${}^{1}$H-^13^C HSQC NMR (Boerkamp et al., 2022)) were used to quantify the lipid oxidation substructures. The TAG glycerol backbone signals were used as internal standard. (C) Example of lipid oxidation substructures in the kinetic model in oxidised trilinolein; other structures were formed in triolein, and trilinolenin. LH: non-reacted lipid,  : alkyl radical,  : peroxyl radical, LOOH: lipid hydroperoxide,  : alkoxyl radical, ALD: aldehyde, KET: ketone, LOH: lipid hydroxide, EP: epoxide, EPOOH: epoxide-hydroperoxide. (D) Schematic representation of model input parameters and how they were used to predict lipid oxidation. The compositional parameters were the initial (t0) concentrations of oleic acid, linoleic acid, alpha-linolenic acid, lipid hydroperoxides (LOOHs), transition metal ions, radical species, antioxidants, and all other oxidation products (*e.g.*, aldehydes). The aldehyde concentration is a function of the oxygen concentration in the oil, the kinetic constant to form aldehydes, and the initial concentration of reactants. The kinetic constant is function of the fatty acid (FA) composition and temperature (T) according to the Arrhenius equation with activation energy $E_{\text{a}}$ and gas constant R.

Lipid autoxidation is a radical chain reaction revolving around lipid hydroperoxides (LOOHs). These molecules are ever present in oils and are said to initiate autoxidation (Fig. 1A) (Laguerre et al., 2020b). When LOOHs degrade by heat or light ($k_{\text{d}}$), reducing metals ($k_{\text{pro2}}$, Fenton reaction), or bimolecular decomposition ($k_{\text{bimol}}$), they form an alkoxyl radical (  ) that converts to secondary oxidation products, amongst others aldehydes ($k_{\text{ald}}$), epoxides ($k_{\text{e1}}$), epoxide-hydroperoxides ($k_{\text{e2}}$), hydroxides ($k_{\text{loh1}},k_{\text{loh2}}$), and ketones ($k_{\text{ket}}$) (Fig. 1A) (Schaich, 2020, Saraev and Pratt, 2024). During this conversion, a hydrogen is abstracted from an unsaturated fatty acid (LH) resulting in an alkyl radical (  ). This highly reactive radical reacts with molecular oxygen ($k_{\text{p1}}$) forming a hydroperoxyl radical (  ).  can abstract a hydrogen from LH ($k_{\text{p2}}$) to form  and LOOH, completing the oxidation cycle. As  is a relatively stable radical with a half-life of 7 s (Pryor, 1984), it can also add to a double bond through a so-called peroxyl radical addition (PRA), which yields an  as well as an epoxide ($k_{\text{e3}}$) or epoxide-hydroperoxide ($k_{\text{e4}}$) (Fig. 1A) (Helberg and Pratt, 2021, Schaich, 2023).

As the formation of the above-described products is relatively slow (weeks to months at room temperature), shelf-life testing of oils and oil-based products typically relies on storage conditions to accelerate oxidation, mostly at elevated temperature. Techniques like oximetry and differential calorimetry can conveniently provide kinetic information (Suhag et al., 2024), but derived rates pertain to lumped reactions (Suhag et al., 2025). The shelf-life at ambient conditions within the supply chain and during consumer use is then predicted using the Arrhenius equation (Calligaris et al., 2016, Farhoosh, 2021). Such approaches often focus on the assessment of only early (Farhoosh, 2021), or late oxidation products (Calligaris et al., 2016), but relying on lumping of down or upstream reactions can lead to erroneous predictions (Calligaris et al., 2016). Furthermore, the fatty acid composition is rarely accounted for in shelf-life prediction models (Farhoosh and Hoseini-Yazdi, 2013, Calligaris et al., 2016, Patsioura et al., 2017, Li et al., 2020, Merkx et al., 2021, Farhoosh, 2022, Schroën and Berton-Carabin, 2022), despite an up to 100-fold difference in oxidation rate amongst common fatty acids (Holman and Elmer, 1947). Hence, current models describing vegetable oil oxidation can only be used to qualitatively describe and rank oxidative stability within their narrow application scope (Farhoosh and Hoseini-Yazdi, 2013, Patsioura et al., 2017, Li et al., 2020, Merkx et al., 2021, Farhoosh, 2022, Schroën and Berton-Carabin, 2022). There is, therefore, a need for predictive mechanistic models that can comprehensively account for environmental conditions, such as temperature, and oxygen partial pressure, as well as compositional factors, such as fatty acid composition, and the initial concentration of antioxidants, prooxidants, hydroperoxides, and radical species. Here, we extended our recently introduced kinetic reaction network model that only partially accounted for environmental and compositional factors (Nguyen et al., 2024b) by fully parametrising temperature and fatty acid composition (Fig. 1A,D). This parametrisation was enabled by our recently developed nuclear magnetic resonance (NMR)-based oxylipidomics platform (Fig. 1B,C) (Merkx et al., 2018, Boerkamp et al., 2022, Boerkamp et al., 2025). This platform quantitatively profiles the main lipid oxidation products of oleic, linoleic and alpha-linolenic acid with a precision that is adequate for constructing and validating kinetic models. The throughput of this platform is also capable of handling the large sample numbers involved in kinetic oxidation studies. In this work, we will parametrise a kinetic network model by accounting for the activation energies of the oxidative reactions of the dominant fatty acids in vegetable oils. For this purpose, we will estimate the kinetic constants of 23 predominant autoxidation reactions for triolein, trilinolein and trilinolenin at different temperatures. We will demonstrate that a parametrised kinetic network model can adequately predict the temperature-dependent formation of primary and secondary oxidation products when applied to showcase applications of vegetable oils in the presence and absence of antioxidants.

## 2 Material and methods

### 2.1 Materials

Triolein (glycerol 9*Z*-octadecanoate), trilinolein (glycerol 9*Z*,12*Z*-octadecadienate), and trilinolenin (glycerol 9*Z*,12*Z*,15*Z*- octadecatrienoate) with 99 % purity were purchased from Larodan (Solna, Sweden). The iron and copper content in the model triacylglycerols (TAGs) was determined by Inductively Coupled Plasma Mass Spectrometry (ICP-MS) (Boerkamp et al., 2024). The iron concentration was (0.16 ± 0.01) mg kg^-1^ for triolein. The iron concentrations in trilinolein and trilinolenin were below the detection limit of 0.1 mg kg^-1^. The copper concentration was below the detection limit of 0.01 mg kg^-1^ for all three TAGs. Rapeseed oil (ADM, the Netherlands) was kindly provided by Unilever (Wageningen, the Netherlands), and sunflower oil was purchased from a local retailer. The fatty acid composition of the rapeseed oil was 6 % saturated, 62 % oleic, 19 % linoleic acid and 8 % alpha-linolenic acid. The saturated fatty acid content consisted of 5 % palmitic acid and 1 % stearic acid. The tocopherol concentration in rapeseed oil was 1 mmol/kg. The iron and copper content of rapeseed and sunflower oil were below the respective detection limits of 0.1 and 0.01 mg kg^-1^ Deuterated chloroform (CDCl_3_) with 0.03 % tetramethylsilane (TMS), deuterated dimethylsulfoxide (DMSO-$d_{\text{6}}$), and deuterated 4 Å molsieves were purchased from Eurisotop (Saint-Aubin, France). Alumina powder (MP EcoChrome Alumina N, Activity: Super I) was purchased from MP Biochemicals (Eschwege, Germany). Tocopherol standards were purchased from Sigma Aldrich (Zwijndrecht, the Netherlands) and the Ultra Performance Liquid Chromatography (UPLC) solvents from Biosolve (Valkenswaard, the Netherlands). All chemicals were used as received without further purification.

### 2.2 Oxidation of model triacylglycerols (TAGs)

Triolein, trilinolein, and trilinolenin were aliquoted (50 mg) into glass vials (internal volume 3.75 mL, internal diameter 14 mm) at 4 °C and 77 % relative humidity (RH) and closed air-tightly with bromobutyl rubber stoppers (Interscience, the Netherlands). In the vials, the column of model TAGs had a height of 0.3 mm. For each TAG and temperature combination, twenty to forty aliquots were incubated in the dark without agitation. The samples were oxidised in the dark to prevent photoxidation. Aliquots were taken as single replicates, as previous work showed low deviations (relative standard deviation below 5 %) (Merkx et al., 2018, Boerkamp et al., 2022). Trilinolenin aliquots were frozen before transferring them to the incubator to increase the reproducibility of its oxidation. Since oxidation rates increase going from triolein, trillinolein to trilinolenin (i.e., with increasing number of double bonds), we could not determine activation energies from kinetic rates at the same set of temperatures. Hence, kinetic rates for triolein, trillinolein and trilinolenin were determined at the respective temperature ranges of 40–70, 25–60 and 4–40 °C. Samples were stored at -20 °C until further use. Oxidised trilinolenin samples were analysed by NMR within one week.

### 2.3 Oxidation of rapeseed oil with endogenous antioxidants

Rapeseed oil was oxidised as previously described (Boerkamp et al., 2022). In short, 1 mL rapeseed oil was aliquoted in clear glass screw-cap vials (internal volume 20.4 mL, internal diameter 20.5 mm). In the vials, the oil column had a height of 3 mm. The samples were oxidised stationary in the dark at 20, 40 and 60 °C. Samples were drawn and stored at -20 °C until further analysis.

### 2.4 Oxidation of stripped sunflower oil

Sunflower oil was stripped from natural antioxidants as previously described (Hoppenreijs et al., 2021). In brief, the oil was mixed with alumina powder (4:1, v/v) in 50 mL centrifuge tubes and rotated head-over-tail overnight at room temperature. Samples were centrifuged at 5000× g for 20 min, after which the oil was decanted and centrifuged at 21,000× g for 20 min to remove excess alumina powder. Stripped oils were stored at -20 °C and used within one week. The fatty acid composition of the stripped sunflower oil was 10 % saturated, 31 % oleic, and 59 % linoleic acid. The saturated fatty acid content consisted of 6 % palmitic acid and 4 % stearic acid. The stripped oil was aliquoted (1 mL) into clear glass screw-cap vials (internal volume 25 mL). We note that for experiments with stripped sunflower oil we turned to vials with 25 mL internal volume since these required less correction for oxygen permeability. In the vials, the oil column had a height of 3 mm. The vials were stored stationary in a dark oven at 25 °C. Duplicates were taken from the oven one to three times a week and stored at -20 °C.

### 2.5 Quantification of fatty acids and lipid oxidation products

Fatty acid composition was determined by gas chromatography coupled to flame ionisation detector (GC-FID) as described previously (Hoppenreijs et al., 2021). Hydroperoxides and aldehydes were quantified by using single pulse and band selective ${}^{1}$H NMR spectra, as previously described (Merkx et al., 2018, Boerkamp et al., 2022). Epoxides, hydroxides, and ketones were quantified by using band-selective 2D ${}^{1}$H-^13^C Heteronuclear Single Quantum Correlation (HSQC) NMR spectroscopy, as previously described (Boerkamp et al., 2022, ten Klooster et al., 2024). The chemical shifts of the epoxides, hydroxides, and ketones were according to our recent work (Boerkamp et al., 2025). A detailed description of these assignments is provided in the supplemental material S1.1. Limit of detection for quantification of hydroperoxides and aldehydes was 0.03 mmol kg^-1^, for an 1D NMR measurement time of 5 min. For all other oxidation products, the limit of quantification was 0.6 mmol kg^-1^, for an 2D NMR measurement time of 70 min. The relative standard deviations for quantification of hydroperoxides and aldehydes ranged between 1%–8%, the higher value corresponding to the limit of quantification. For all other oxidation products, the relative standard deviations ranged between 1%–10%. The relative standard deviations correspond to reproducibility, which includes day-to-day variation (Merkx et al., 2018, Boerkamp et al., 2022).

### 2.6 Quantification of tocopherol

Tocopherol was quantified by Reverse Phase UPLC/Photodiode Array (RP-UPLC/PDA) based on previous work (Moltó-Puigmartí et al., 2009, Plankensteiner et al., 2024). A detailed description is provided in the supplemental material S1.2.

### 2.7 Kinetic modelling

#### 2.7.1 Reaction network design

We started from the principle that a simpler kinetic network is a better network. The kinetic model presented in this work builds on our recent quantitative kinetic model for oil autoxidation (Nguyen et al., 2024b). Ketones and hydroxides (Eq. (8), (9), (10)) were included as they are predominant in the oxidation of triolein (Boerkamp et al., 2025). For the formation of hydroxides, initially, only the hydrogen abstraction from LH to form hydroxides was included (  with kinetic constant $k_{\text{loh1}}$). However, a network with only $k_{\text{loh1}}$ to form hydroxides lead to poor fits of the hydroxide (LOH) concentration. In contrast, good fits (normalised root mean square error, NRMSE < 0.05) were obtained by adding $k_{\text{loh2}}$ in which  abstracts a hydrogen from LOOH (Laguerre et al., 2020a). During the formation reaction of ketones, aldehydes, and epoxides,  could also abstract a hydrogen from LOOH instead of LH. As the model performance without these reactions was good in all of our tests, these reactions were not included to keep the model as simple as possible. Peroxyl radical addition reactions (Eq. (13), (14)) were added, as these are crucial propagation reactions (Helberg and Pratt, 2021, Schaich, 2023). The radical recombinations, which terminate lipid oxidation radical chains, were included to make the model complete (Eq. (19), (20), (21)). The rate constants of these reactions were not sensitive, which means that the exact value of the rate constants did not significantly change the goodness of the fit (Nguyen et al., 2024b). Finally, to predict lipid oxidation in the presence of antioxidants (AH), reactions between AH and the radicals  , and  were considered (Eq. (16), (17), (18), (22), (23)). The resulting reactions are listed below and connected together in a kinetic network (Fig. 1D). In these reactions, each term ‘***k***’ embodies a vector of kinetic constant with one entry for each type of fatty acid, i.e., k=[k(triolein),k(trilinolein),k(trilinolenin)]. NRP stands for non-radical products.

(1)
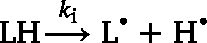

(2)
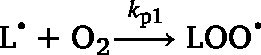

(3)
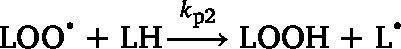

(4)
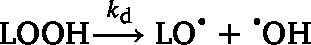

(5)
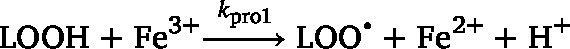

(6)
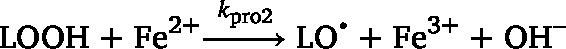

(7)
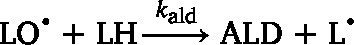

(8)
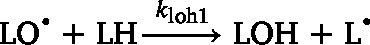

(9)
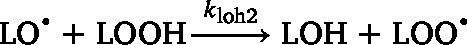

(10)
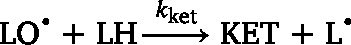

(11)
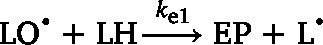

(12)
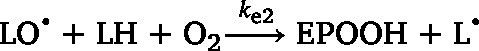

(13)
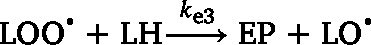

(14)
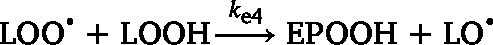

(15)
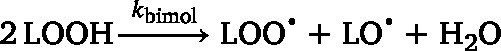

(16)
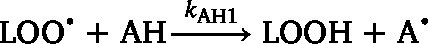

(17)
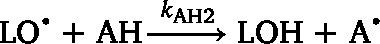

(18)
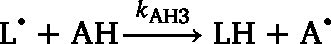

(19)
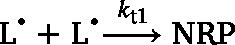

(20)
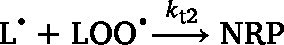

(21)
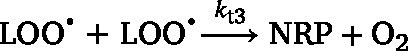

(22)
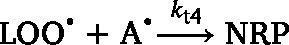

(23)
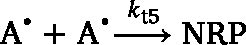

#### 2.7.2 Model construction

In the model, the kinetic rates of the six main oxidation products (*i.e.*, LOOH, ALD, LOH, KET, EP, and EPOOH), and tocopherols were expressed as the sum of the kinetic rates of the relevant underlying reactions (Eqs. (1), (2), (3), (4), (5), (6), (7), (8), (9), (10), (11), (12), (13), (14), (15), (16), (17), (18), (19), (20), (21), (22), (23)).

(24) $$\frac{\text{d}\left[\mathrm{LOOH}\right]}{\text{d}t}=r_{\text{p2}}-\left(r_{\text{d}}+r_{\text{pro1}}+r_{\text{pro2}}+r_{\text{loh2}}+r_{\text{e4}}+r_{\text{bimol}}\right)+r_{\text{AH1}}$$

(25) $$\frac{\text{d}\left[\mathrm{ALD}\right]}{\text{d}t}=r_{\text{ald}}$$

(26) $$\frac{\text{d}\left[\mathrm{LOH}\right]}{\text{d}t}=r_{\text{loh1}}+r_{\text{loh2}}+r_{\text{AH2}}$$

(27) $$\frac{\text{d}\left[\mathrm{KET}\right]}{\text{d}t}=r_{\text{ket}}$$

(28) $$\frac{\text{d}\left[\mathrm{EP}\right]}{\text{d}t}=r_{\text{e1}}+r_{\text{e3}}$$

(29) $$\frac{\text{d}\left[\mathrm{EPOOH}\right]}{\text{d}t}=r_{\text{e2}}+r_{\text{e4}}$$

(30) $$\frac{\text{d}\left[\mathrm{AH}\right]}{\text{d}t}=r_{\text{AH1}}+r_{\text{AH2}}+r_{\text{AH3}}$$

(31)
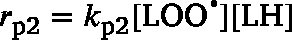

(32)
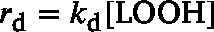

(33)
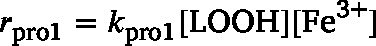

(34)
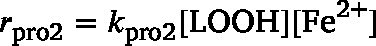

(35)
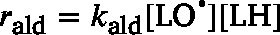

(36)
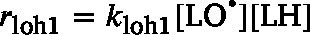

(37)
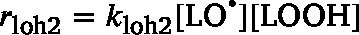

(38)
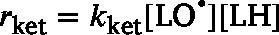

(39)
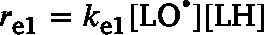

(40)
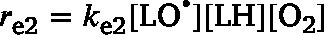

(41)
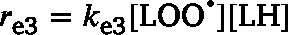

(42)
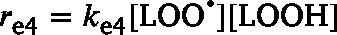

(43)
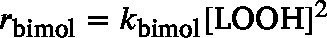

(44)
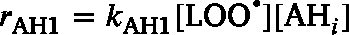

(45)
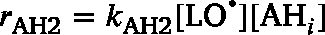

(46)
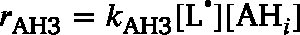

The radicals, and oxidation products were assumed to be uniformly distributed in the headspace and oil phase. The concentrations in Eq. (24), (25), (26), (27), (28), (29), (30) are the concentrations in the oil. In the absence of antioxidants (*i.e.*, model TAGs and in stripped oils), Eq. (30) was not considered, which means that d[AH]/dt=0.

Due to the high reactivity of trilinolenin, oxidation was observed even during NMR acquisition. We hypothesised the formation to be proportional to the LOOH concentration. Thus, the Ordinary Differential Equations (ODEs) for aldehydes (Eq. (25)) and hydroxides (Eq. (26)) were modified to:

(47) $$\frac{\text{d}{\left[\mathrm{ALD}\right]}_{\text{trilinolenin}}}{\text{d}t}=r_{\text{ald}}+\alpha_{\text{ALD}}\cdot \frac{\text{d}\left[\mathrm{LOOH}\right]}{\text{d}t}$$

(48) $$\frac{\text{d}{\left[\mathrm{LOH}\right]}_{\text{trilinolenin}}}{\text{d}t}=r_{\text{loh1}}+r_{\text{loh2}}+r_{\text{AH2}}+\alpha_{\text{LOH}}\cdot \frac{\text{d}\left[\mathrm{LOOH}\right]}{\text{d}t}$$

where $\alpha_{\text{ALD}}$ and $\alpha_{\text{LOH}}$ are estimated constants that determine the formation rate during NMR acquisition, and $\frac{\text{d}\left[\mathrm{LOOH}\right]}{\text{d}t}$ was the rate of LOOH formation (Eq. (24)). The constants were assumed to be independent of oxidation storage conditions (*i.e.*, accelerated shelf-life temperature). These constants were estimated by determining the formation of aldehydes and hydroxides in an NMR sample (150 μL oil and 450 μL CDCl${}_{3}$:DMSO 5:1). By fitting the formation to Eq. (47), (48), we estimated $\alpha_{\text{ALD}}$ = 0.0018 and $\alpha_{\text{LOH}}$ = 0.0025. Eq. (47), (48) were only used for the oxidation of trilinolenin, and not for the oxidation of other model TAGs, fatty acid mixtures, or oils.

#### 2.7.3 Model assumptions

The following assumptions were made to construct the model in this study.

1. The oxidation system (oil and vial) was assumed as a two-phase (oil and headspace) system (Fig. 1A) (Nguyen et al., 2024b).
2. Oxygen, radicals, and oxidation products were assumed to be uniformly distributed in the oil phase (Nguyen et al., 2024b). The assumption was based on estimated diffusion constants of 1–2.5 x 10^-9^ m^2^ s^-1^ for the temperature range 20–60 °C (Nguyen et al., 2026, Pénicaud et al., 2010) , and the height of the oil columns in the vials.
3. Oxygen was assumed to be uniformly distributed in the headspace (Nguyen et al., 2024b).
4. The kinetic constant of Fe^3＋^ (Eq. (5)) was assumed to be 100 times lower than the kinetic constant of Fe^2＋^ (Eq. (6)) to reduce possible redundancy in the model parameters (Nguyen et al., 2024b, Choe and Min, 2006).
5. The initial concentration of radicals was assumed to be equal to the formation rate at initial time t0. For instance, the formation rate of  is the sum of the kinetic rates of reactions in Eq. (2), (5), (9), (15). At initial time, the formation of  related to reactions in Eq. (9), (15) is negligible. In our model, we calculated the initial concentration of  based on only reactions in Eq. (2), (5):  , where ${\left[\mathrm{LH}\right]}_{\text{t0}}$, ${\left[O_{2}\right]}_{\text{t0}}$, ${\left[\mathrm{LOOH}\right]}_{\text{t0}}$, and ${\left[{\mathrm{Fe}}^{3+}\right]}_{\text{t0}}$ are the concentrations of LH, O_2_, LOOH, and Fe^3＋^ in the bulk oil at initial time t0 and given from experimental data.

#### 2.7.4 Oxygen mass transfer

The model included O_2_ mass transfer incorporated explicitly with the dimensions of vials and oil samples, which allowed accounting for changes in headspace-to-oil ratio robustly in the model. At the headspace-oil surface, O_2_ from the headspace is dissolved in the bulk oil (denoted by $\varphi^{\text{oil}}$). The initial O_2_ partial pressure in the headspace was created when the container was sealed. At the headspace-oil surface, O_2_ from the headspace dissolved in the bulk oil until an equilibrium is reached at the O_2_ solubility limit. During oxidation, headspace oxygen reflects two processes: diffusion from headspace into oil as lipids in the oil take up the dissolved O_2_ (denoted by $\varphi^{\text{s}}$), and diffusion of O_2_ into the headspace from the surrounding atmosphere *via* permeation through the cap plastic or leakage through the cap connection. Kinetic models must account for both processes. The O_2_ mass transfer processes in the two phases were expressed as:

(49) $$\frac{\text{d}\left[{\text{O}}_{2,\text{HS}}\right]}{\text{d}t}=\frac{1}{V_{\text{HS}}}\left(\varphi_{{\text{O}}_{2}}^{\text{s}}+\varphi_{{\text{O}}_{2}}^{\text{Pe}}\right)$$

(50) $$\frac{\text{d}\left[{\text{O}}_{2,\text{oil}}\right]}{\text{d}t}=\frac{1}{V_{\text{oil}}}\left(\varphi_{{\text{O}}_{2}}^{\text{s}}+\varphi_{{\text{O}}_{2}}^{\text{oil}}\right)$$

where $\varphi^{\text{Pe}}$, $\varphi^{\text{oil}}$, and $\varphi^{\text{s}}$ (in mol s^-1^) describe the kinetic rate of the change in O_2_ concentration through the cap (*i.e.*, permeability), at the oil-headspace surface, and in the oil phase, respectively. $V_{\text{HS}}$ is the volume of the headspace and $V_{\text{oil}}$ the volume of the oil. The estimation of $\varphi^{\text{Pe}}$, $\varphi^{\text{surf}}$, and $\varphi^{\text{s}}$ is further detailed in our previous work (Nguyen et al., 2024b).

#### 2.7.5 Global optimisation

The kinetic constants k in Eq. (24), (25), (26), (27), (28), (29), (30) were estimated by fitting the LOOH, ADs, EPOOHs, EPs, KETs and LOHs data over time. To simultaneously fit the curves X with the multiple experimental results, we employed a multi-response optimisation (Nguyen et al., 2024b) using Matlab R2021b (Mathworks, Natick, MA, USA). The ODE system in Eq. (24), (25), (26), (27), (28), (29), (30) was numerically solved by the ‘ode15s’ solver. Then, kinetic constants k were determined by minimising the sum of the squared errors between experimental data and model outcomes by the ‘lsqnonlin’ algorithm. In our dataset, the concentrations of oxidation products differed in orders of magnitude. For example, in our data of trilinolein at four temperatures, the LOOH concentration varied between 0 and 600 mmol kg^-1^ oil, whereas the concentration of EPs, EPOOHs varied between 0 and 60 mmol kg^-1^ oil, and ALDs, KETs, and LOHs varied between 0 and 20 mmol kg^-1^ oil. To equally weight all products in the optimisation procedure, the respective datasets ($\overset{˜}{X}$) were normalised between 0 and 1, following Eq. (51), before calculating the sum of squared errors (Eq. (52)).

Kinetic constants from others studies were estimated using a different (simplified) scheme of lipid oxidation reactions and models other than the present model. Therefore, the kinetic constants in our model were not expected to be identical. Literature values were, however, used to set the initial values of model parameters included in the optimisation procedure. As each set of starting values returned different estimates, the optimal estimates were selected based on the least estimate of errors (Eq. (52)).

(51) $${\overset{˜}{X}}_{i}=\frac{X_{i}-\min\left(X_{exp}\right)}{\max\left(X_{exp}\right)-\min\left(X_{exp}\right)}$$

where subscript i denotes either a numerical or experimental curves, and exp represents experimental kinetic curves.

(52) $${\left\|{\overset{˜}{X}}_{exp}-{\overset{˜}{X}}_{num}\right\|}_{2}^{2}=\overset{n_{t}}{\underset{i=1}{\sum }}{\left(\overset{˜}{X}{\left(t_{i}\right)}_{exp}-\overset{˜}{X}{\left(t_{i}\right)}_{num}\right)}^{2}$$

where t indicates the storage time, i the time point index, and $n_{t}$ the total number of time points. After fitting the numerical and experimental datasets, the precision of the estimates was evaluated using Monte Carlo simulations with 200 iterations to calculate the standard deviation (Nguyen et al., 2024b). As in this previous work, we determined the standard deviation as $X_{\text{noise,exp}}=X_{\text{exp}}$ + σ × r${}_{1}$ × r${}_{2}$, where $X_{\text{noise,exp}}$ and $X_{\text{exp}}$ are the noised and original experimental data, respectively; σ is the experimental standard deviation estimated for each experimental data; r${}_{1}$ and r${}_{2}$ are random values from uniform distribution between 0 to 1. Then, we applied the Box–Muller algorithm to the calculation of r${}_{1}$ × r${}_{2}$, which allowed to generate 200 random values following Gaussian distribution between 0 and 1 without bias distribution (Hessler, 1997). Thus, Monte Carlo simulations combined with the Box–Muller algorithm guaranteed that 200 iterations are sufficient to ensure parameter robustness (Coffigniez et al., 2018).

Once the estimates were determined, they were used as reference values in local sensitivity analysis (SA) tests. In the local SA tests, we adjusted each kinetic constant by multiplying with a factor varying between 1/1000 to 1000. Then, we calculated the NRMSEs between the model outcomes derived using the reference estimates and the adjusted estimates. Some reactions (*e.g.*, $k_{\text{d}}$ for 18:1, and 18:3) were insensitive, meaning that model outcomes derived using the adjusted estimates were close to the model outcomes derived using the reference estimates (NRMSEs < 0.05).

#### 2.7.6 Activation energy estimation

The log form of Arrhenius equation was expressed as linear equation Y=aX+b in which $Y=\ln\left(k/k_{\text{ref}}\right)$, a=−1/R, $X=E_{\text{a}}$, and b = $\left(\frac{1}{T}-\frac{1}{T_{\text{ref}}}\right)$, where R is the gas constant, and ref stands for reference. The lowest incubation temperature was selected as reference temperature. Finally, the activation energy $E_{\text{a}}$ was estimated by satisfying $E_{\text{a}}=X\approx \left(Y-b\right)/a$. The Y values are displayed in the supplementary material, Table S1 (k values for triolein), Table S2 (k values for trilinolein), and Table S3 (k values for trilinolenin) at four different temperatures.

#### 2.7.7 Kinetic rate constants in a mixture of TAGs with varying fatty acids

By fitting the model to the data of the oxidised model TAGs (triolein, trilinolein, and trilinolenin), the kinetic constants for all reactions of oleic (Table S1), linoleic (Table S2), and alpha-linolenic (Table S3) acid residues were estimated. The model fitting did not estimate the cross-kinetic constants, *e.g.*, in propagation reactions between  (oleic) and LH(linoleic). Thus, as a step towards applying the model to generate predictions of lipid oxidation products in vegetable oils, we estimated the cross-kinetic constants based on kinetic constants of model TAGs, similar as described previously for fatty acid methyl esters (FAMEs) (Touffet et al., 2023). It was reported that the hydrogen abstraction rate does not depend on the radical molecular structure but on the hydrogen donor (Touffet et al., 2023). Therefore, the kinetic constant $k_{\text{p2}}$ of reaction between  (18:2) and LH(18:1), for example, (53)
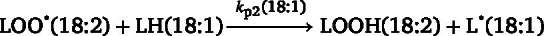
was assumed to be equal to the $k_{\text{p2}}$ of the reaction between  (18:1) and LH(18:1). Here, 18:2 stands for a linoleic acid residue and 18:1 for an oleic acid residue. In other words, the reaction rate constants for reaction between  (18: i) and LH(18: j) where i and j designate the number of double bonds were assumed to be $k_{\text{p2}}$(18: j).To make calculation easier, this assumption was also true for reactions between other radicals and LH(18: j).

In a similar fashion, when EPOOHs were formed by  (Eq. (12)), the cross-kinetic constant was approximated to the $k_{\text{e2}}$ of the LH. (54)
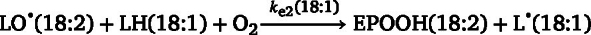


For the formation of EPOOH by a peroxyl radical addition of  on an LOOH, the kinetic constant ($k_{\text{e4}}$) of the LOOH species was used. (55)
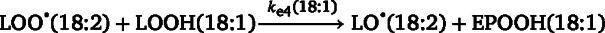


Similarly, for the formation of hydroxides (LOH) and ketones (KET), the kinetic constant of the hydrogen donor (LH) was used.

(56)
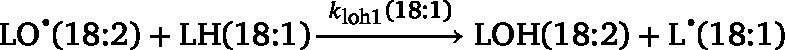

(57)
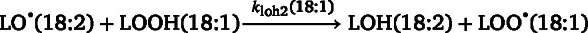

(58)
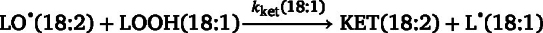

On the other hand, for $k_{\text{e1}}$ and $k_{\text{e3}}$, the kinetic constant of the radical species was used, as the cyclisation rate was assumed to be dependent on the radical species (Schaich, 2020). (59)
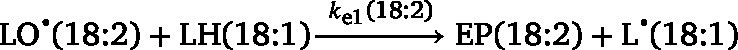

(60)
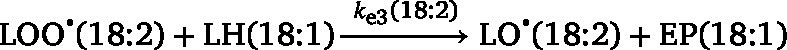


### 2.8 Model prediction for oxidation of oil without antioxidants

We constructed a kinetic model based on the activation energies of the predominant unsaturated fatty acids in a vegetable oil determined in pure TAGs containing only one type of FA. The model is then used to predict the oxidation of stripped sunflower oil under different temperature conditions and antioxidant concentrations. Fatty acid composition and shelf-life conditions, such as storage time and vial dimensions were used as model input. The values of kinetic constants k at 30 °C were interpolated using the Arrhenius equations with activation energies in Table 1, and plugged into the model to simulate the formation of oxidation products. We stress that we did not finetune, fit or train our model to the experimental data of vegetable oils. The model generated predictions based on a model that was parametrised on the basis of model TAGs. The cross-kinetic constants were incorporated as described in the section above.

**Table 1:** Activation energies ($E_{\text{a}}$, in kJ mol^-1^) of triolein, trilinolein and trilinolenin. The relative standard deviation was less than 1 % as determined by Monte Carlo simulations. Activation energies were not estimated for $k_{\text{pro1}}$, $k_{\text{pro2}}$, and $k_{\text{loh1}}$ as the model output was insensitive to these kinetic constants.

	Triolein	Trilinolein	Trilinolenin
$k_{i}$	2452	701	35
$k_{\text{p1}}$	4.5	23	19
$k_{\text{p2}}$	12	8.0	11
$k_{\text{d}}$	*n.d.*^a^	95	*n.d.*^a^
$k_{\text{bimol}}$	63	76	86
$k_{\text{e1}}$	29	95	*n.d.*^b^
$k_{\text{e2}}$	*n.d.*^b^	28	131
$k_{\text{e3}}$	30	23	*n.d.*^b^
$k_{\text{e4}}$	*n.d.*^b^	63	107
$k_{\text{ald}}$	49	161	16
$k_{\text{loh2}}$	7.6	29	7.8
$k_{\text{ket}}$	2.8	42	*n.d.*^b^

a Not determined (n.d.) as k was insensitive, *i.e.*, changing k by a factor 1/1000 to 1000 did not change the goodness of the fit.

b Not determined (n.d.) due to low product concentration.

#### 2.8.1 Modelling oxidation of oil with antioxidants

A rapeseed oil with an endogenous tocopherol concentration of 1 mmol/kgoil was used. As the storage temperatures of the oil differed from the storage temperature of the model TAGs, the kinetic constants ***k*** were extrapolated and interpolated (to 20, 40 and 60 °C) using the Arrhenius equations with activation energies in Table 1. Although the initial concentration of endogenous tocopherols is low, in the range of µM, tocopherols play an important role on the induction period of LOOH formation, and thus required to evaluate the antioxidant activity.

The same model was applied as in Section 2.7.7, only the kinetic constants of tocopherols, $k_{\text{AH1}}$, $k_{\text{AH2}}$, $k_{\text{AH3}}$, $k_{\text{t4}}$, and $k_{\text{t5}}$, were estimated. To do so, only three time points during early short-term formation of LOOH (Eq. (24)) were used. In this time frame, LOOH concentrations did not exceed 10 mmol kg^-1^. The estimated values of ***k***, and other values given from experiments such as initial concentrations of UFAs and oxidation products, and vial dimensions were plugged into the model to predict the formation of oxidation products.

When applied to predict for oils at 20 °C, the second acceleration phase was taken into consideration by estimating $C_{\text{LOOH,critical}}$ and the multiplication factors f (as provided in Table S4). In our current test, aside from multiplication factors f, we also included a critical LOOH concentration ($C_{\text{LOOH,critical}}$) in the list of parameters to be estimated. The values of $C_{\text{LOOH,critical}}$ in our previous study on different vegetable oils ranged from 80 to 95 mmol kg^-1^ oil and were thus used as starting values for the optimisation (Nguyen et al., 2024b). Thus, after LOOHs reached $C_{\text{LOOH,critical}}$, the second acceleration was fitted using $k_{C_{\text{LOOH,critical}}}=f\cdot k$.

## 3 Results and discussion

### 3.1 Estimation of activation energies in model triacylglycerols

**Fig. 2:**
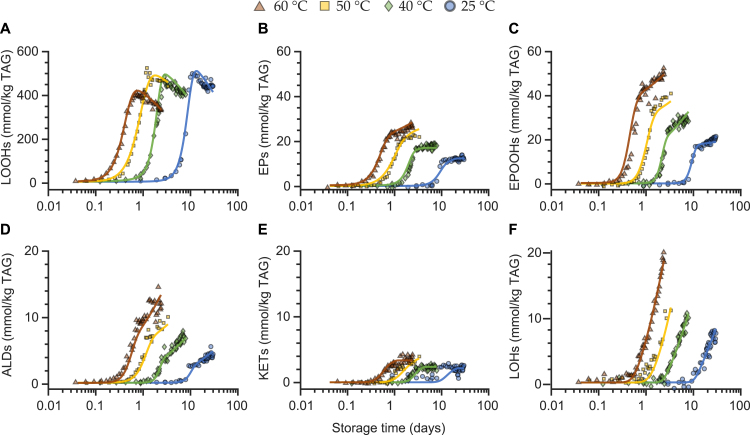
Estimating the kinetic constants of oxidation reactions in trilinolein at four temperatures. The concentration upon oxidation of lipid (A) hydroperoxides (LOOHs), (B) epoxides (EPs), (C) epoxide-hydroperoxides (EPOOHs), (D) aldehydes (ALD), (E) ketones (KETs), and (F) hydroxides (LOHs). The circles are experimental data and lines are outcomes of global fits.

In order to obtain a model that can predict vegetable oil oxidation at different temperatures, the activation energies for the reactions in the kinetic network first need to be estimated for each relevant fatty acid (Fig. 1A). Hereto, we autoxidised three model triacylglycerols (TAGs) in the dark at four temperatures. The model TAGs consist of a glycerol backbone and one of the three dominant fatty acids in vegetable oils (oleic, linoleic, alpha-linolenic acid). During autoxidation, lipid hydroperoxides, epoxides, epoxide-hydroperoxides, hydroxides, ketones, and aldehydes were quantified by NMR spectroscopy (Fig. 1B,C) (Merkx et al., 2018, Boerkamp et al., 2022, Boerkamp et al., 2025). These products together accounted for approximately 95 % of reacted substrate (supplementary material, Figure S2). The obtained concentration profiles were used to estimate all the reaction kinetic constants in our kinetic network (Fig. 1A). The kinetic rates to form each product class were described as ordinary differential equations (ODEs, Eq. (24), (25), (26), (27), (28), (29), (30)). The experiments were performed in vials, where the oil column has a height of 0.3 mm. Assuming diffusion constants of 1–2.5 × 10^-9^ m^2^ s^-1^ for the temperature range 20–60 °C, (Nguyen et al., 2026), (Pénicaud et al., 2010) , we can estimate that oxygen will be homogeneously distributed in the oil phase at the minute scale. Thus, we can further estimate that oxygen diffusion at this time scale occurs faster than lipid oxidation reactions in our experiments. As a consequence, also radicals and oxidation products will be homogeneously distributed. Therefore, as in previous work, we considered the vials as two-phase oil-headspace systems (Nguyen et al., 2024b). Mass transfer of oxygen between headspace and oil phase was accounted for, as previously described (Nguyen et al., 2024b). We included equations related to oxygen mass transfer (Eq. (49), (50)) to account for the ratio between headspace surface area and oil volume, the headspace volume, the oxygen permeability through the container, and the oxygen solubility in the oil. The presence of catalytically active transition metal was also considered for in the reaction network model for triolein, trilinolein and trilinolenin. The iron concentration was 0.16 mg kg^-1^ for triolein and below the detection level of 0.1 mg kg^-1^ for trilinolein, and trilinolenin. No copper could be detected. In line with previous work (Nguyen et al., 2024b) (3), we assumed a ratio of 1/100 between the rates of the reactions where ferric (Fe^3＋^) and ferrous (Fe^2＋^) ions are involved. The rate constants for the initiation reaction (Eq. (1), k${}_{1}$) and catalytic reaction (eq (6), $k_{\text{pro2}}$), were estimated in previous work (Nguyen et al., 2024b). The kinetic constants of all reactions (Eq. (1), (2), (3), (4), (5), (6), (7), (8), (9), (10), (11), (12), (13), (14), (15), (16), (17), (18), (19), (20), (21), (22), (23)) were estimated by fitting the formation of all oxidation products simultaneously using a global multi-response optimisation method. The optimisation yielded high quality model fits at each oxidation temperature (Fig. 2, and Figures S3 and S4 in the supplementary material). The estimated rate constants were not sensitive to iron concentration in the 0–0.2 mg kg^-1^ range. This indicates that at these low iron concentrations, initiation reaction (Eq. (1), k${}_{1}$) is dominant over the catalytic reaction (Eq. (6), $k_{\text{pro2}}$). The estimated kinetic constants at varying temperature enabled us to determine the activation energy for each reaction (Fig. 2, and Figures S3 and S4). The activation energies were estimated by fitting the classical Arrhenius equation to the kinetic constants at four temperature points (supplementary material, Figure S1, Table 1). Using these estimated activation energies for triolein, trilinolein, and trilinolenin, we were able to parametrise temperature and fatty acid composition in the reaction network (Fig. 1A) and thus predict the formation of oxidation products of vegetable oils at different temperatures.

### 3.2 Construction of a kinetic model for vegetable oils

As for the model TAG, the experiments for vegetable oils were performed in vials, where the oil column had a height of 3 mm. We estimated that oxygen will homogeneously distribute itself on a time scale of hours. Also at this time scale, oxygen diffusion is faster than lipid oxidation in our experiments. Hence, also the vials with vegetable oils can be considered as two-phase oil-headspace systems. Mass transfer of oxygen between headspace and oil phase and limited permeability of the vial cap for oxygen were accounted for in a similar manner as for the model TAG vials (Nguyen et al., 2024b). In line with previous work, we estimated starting concentrations of radicals from their initial formation rates (Nguyen et al., 2024b). The parametrisation for temperature and fatty acid composition, based on the oxidation of model TAGs, only accounts for self-reactions between the same fatty acid residues. For example, reactions of oleate residue with another oleate residue are captured, but not cross-reactions between different fatty acid residues (*e.g.*, an oleate residue with a linoleate or alpha-linolenate residue). Recently, it was reported that the hydrogen abstraction rate does not depend on the radical molecular structure but on the hydrogen donor (Touffet et al., 2023). Therefore, when considering the hydrogen abstraction by a triolein  from an non-reacted trilinolein (LH), the kinetic constant $k_{\text{p2}}$ of trilinolein was used. Similarly, when a triolein  was added to the double bonds of trilinolein, the $k_{\text{e1}}$ of trilinolein was used. The same logic was used for all other reactions, as further detailed in Section 2.7.7. For all vegetable oils studied here, copper concentrations were below 0.01 mg kg^-1^ and thus excluded from further consideration. Hence, only the presence of iron, for which the concentration was below 0.1 mg kg^-1^, was considered in the model applications for vegetable oils (Choe and Min, 2006).

### 3.3 Application of the kinetic model to a vegetable oil stripped of antioxidants

**Fig. 3:**
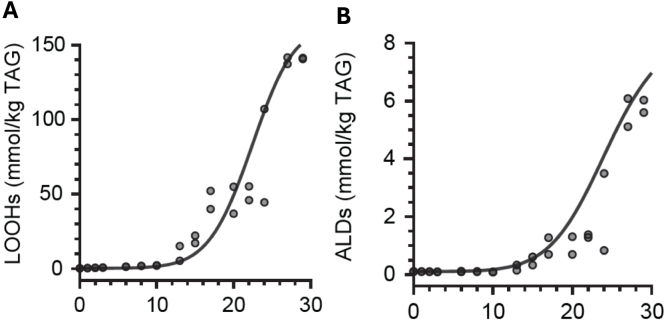
Prediction of lipid hydroperoxide and aldehyde formation in stripped sunflower oil. Plot (A) and (B) respectively show hydroperoxide (LOOH) and aldehyde (ALD) formation. The oil contained 10 % saturated, 31 % oleic, and 59 % linoleic acid and was oxidised in the dark in 25 mL vials at 40 °C. Replicates are plotted as individual data points.

We evaluated the performance of the constructed model to predict lipid oxidation in a sunflower oil stripped of its natural antioxidants and oxidised at 40 °C. The oxidation of this sunflower oil was predicted using the activation energies (Table 1) in combination with known environmental (vial dimensions, oxygen concentration, temperature) and compositional parameters (initial LOOH concentration, fatty acid composition, metal concentration, estimated radical species concentration). The resulting predicted concentrations of LOOHs and aldehydes agreed well with the experimental data (Fig. 3A,B), which indicates a successful parametrisation by temperature and fatty acid composition. In the model, the concentration of iron was varied in the range of 0–0.2 mg kg^-1^, without resulting in significantly different outcomes. We concluded that at these low iron concentrations, initiation reaction (Eq. (1), k${}_{1}$) is dominant over the catalytic reaction (Eq. (6), $k_{\text{pro2}}$).

### 3.4 Application of the kinetic model to a vegetable oil with endogenous antioxidant

Finally, the model was applied to a commercial rapeseed oil with endogenous tocopherol present. Radical scavenging antioxidants, such as tocopherols, compete with the previously described lipid oxidation reactions by donating a hydrogen atom to lipid radicals, such as  , where AH is an antioxidant. During this reaction, no new lipid radical  is formed, thereby breaking a radical chain (Helberg and Pratt, 2021). Furthermore, the antioxidant radical  can trap peroxyl radicals by  (Helberg and Pratt, 2021). Due to these two modalities, every tocopherol molecule breaks two radical chains (Pratt et al., 2001). Thus, to be able to model lipid oxidation in the presence of antioxidants, the network was extended with reactions between hydrogen donating antioxidants and lipid radicals (  ,  , and  ) (Eq. (16), (17), (18)), as well as with reactions between antioxidant radicals and lipid radicals (Eq. (22), (23)).

**Fig. 4:**
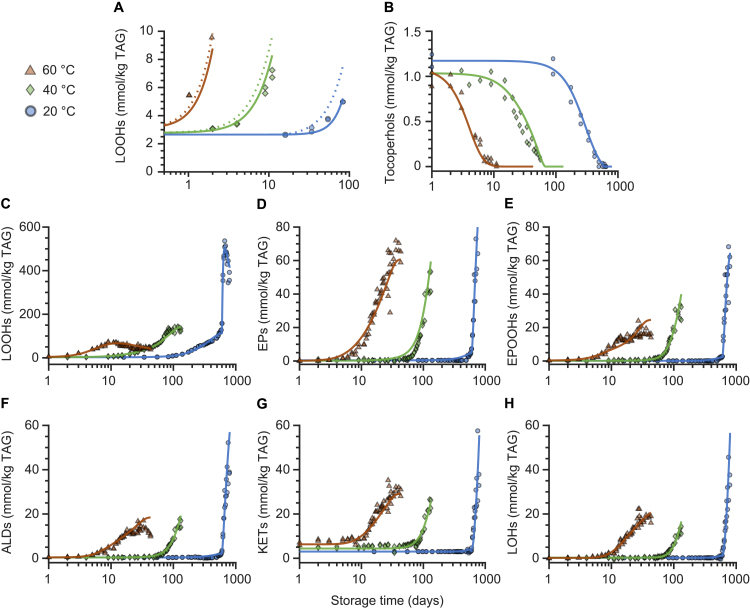
Modelling the oxidation kinetics of rapeseed oil with endogenous tocopherols as antioxidant. (A) Estimating antioxidant efficacy by fitting the early LOOH formation. The dotted line shows the prediction of LOOH formation without antioxidants and the solid line shows the LOOH formation with antioxidants after estimating the antioxidant kinetic constants (Eq. (22), (23), (44), (45), (46)). (B) The prediction of tocopherols degradation using the kinetic constants determined in panel A. The estimated kinetic constants of tocopherol in panel A were also used to predict the concentration upon oxidation of lipid (C) hydroperoxides (LOOHs), (D) epoxides (EPs), (E) epoxide-hydroperoxides (EPOOHs), (F) aldehydes (ALDs), (G) ketones (KETs), and (H) hydroxides (OHs). The circles are experimental data, and the lines are predictions generated by the model. The oil (1 mL) was stored in the dark in closed vials (20 mL). The prediction of oxidation at 20 °C from 580 days onwards was generated by multiplying the kinetic constants with an acceleration factor (Table S4).

The modelling of lipid oxidation in the presence of antioxidants was performed in three steps: (i) estimation of the kinetic constants of reactions with tocopherol, (ii) validation of the estimated kinetic constants, and (iii) prediction of a vegetable oil oxidation using the estimated kinetic constants. We estimated the kinetic constants of endogenous tocopherols (Eq. (16), (17), (18), (22), (23)) in rapeseed oil. Hereto, the rapeseed oil was oxidised at 20, 40, and 60 °C; the oxidation products were quantified over time by NMR, and the tocopherols by UPLC-PDA. The antioxidant efficacy was estimated by only fitting the early stage of hydroperoxide formation (Fig. 4A) in combination with the previously determined oxidation kinetic constants of the model TAGs (Tables S1–S3, supplementary material). The estimated kinetic constants of tocopherols (Table S5, supplementary material) were then validated by predicting the degradation of tocopherols over a longer period. The predicted tocopherol concentrations were in excellent agreement with experimental data (Fig. 4B), which shows that the antioxidant kinetic constants, and therefore the antioxidant efficacy, can be adequately determined using only the early stage of LOOH formation.

The estimated kinetic constants and the initial concentration of tocopherols enabled the prediction of rapeseed oil oxidation with antioxidants as previously described for the stripped oils (Fig. 3). The predicted formation of oxidation products matched well with the experimental data (Fig. 4C–H), in particular at 40 °C and 60 °C. However, at 20 °C, from 580 days onwards, a slower oxidation rate was predicted than experimentally observed (Figure S5). After 580 days, the experimental data showed an acceleration in the oxidation rate, as previously reported in vegetable oils when a critical concentration of lipid peroxides is reached (Farhoosh, 2018). At such a critical concentration of hydroperoxides, micellar structures can be formed, which brings hydroperoxides and other reactants in closer proximity (Nuchi et al., 2002, Chen et al., 2012, Budilarto and Kamal-Eldin, 2015, Laguerre et al., 2020b, Villeneuve et al., 2021). In vegetable oils, reverse micelle formation is enhanced by the presence of surface active compounds, such as phospholipids, and diacylglycerols (Nuchi et al., 2002, Chen et al., 2012, Villeneuve et al., 2021). This explains why for the pure model TAGs no acceleration was observed (Fig. 2 and Figure S3, and S4). Our data points to a critical concentration of approximately 113 mmol/kg TAG for rapeseed oil at 20 °C, which is comparable to previous estimates (Ghnimi et al., 2017, Farhoosh, 2018, Khabbaz et al., 2023). By estimating the accelerated reaction rates above the critical concentration (Table S4) (Nguyen et al., 2024b)(Merkx et al., 2021), lipid oxidation was adequately predicted (Fig. 4C-H). As hydroperoxides degrade quickly at 40 and 60 °C, the critical LOOH concentration was not reached and no acceleration was observed at those temperatures.

We realise that our reaction network approach only considers the chemical events during autoxidation and that at critical concentrations of reactants, also colloidal mechanisms come into play, which lead to significant acceleration of oxidation rates (Villeneuve et al., 2021). Experimental approaches to directly assess these temperature dependent critical concentrations are lacking but literature estimates are in the range of 50–200 mM (Ghnimi et al., 2017, Farhoosh, 2018, Khabbaz et al., 2023, Nguyen et al., 2024b). By our current reaction network model, we can estimate the time at which critical reactant concentrations are reached, and thus provide a lower limit for shelf-life. Typically, shelf-life is determined at accelerated conditions; the parametrised model can help to extrapolate the shelf-life at elevated temperature to ambient conditions, thus reducing trial requirements. We also note that the scope of our current reaction network is limited to oleic, linoleic and alpha-linolenic acid as the main fatty acids in vegetable oils. Our approach can also be applied to oils comprising other polyunsaturated fatty acids. This will involve extending the oxylipidomics platform (Boerkamp et al., 2025) with assignment of ${}^{1}$H NMR signals of corresponding oxidation products. As in our current work, this will be most convenient for triglycerides comprising only a single polyunsaturated fatty acid. Next, the reaction network can be used to determine kinetic rates at different temperatures. Subsequently, the activation energies can be determined for the reactions in the network. This will then enable model parametrisation for a broader range of edible oils. We also envisage extension of our parametrised model to food emulsions where interfacial effects come into play (Hennebelle et al., 2024). Current partially parametrised models have already been extended to emulsions (Nguyen et al., 2024a) by accounting for descriptors of both colloidal structures and reaction networks. The latter can now also be fully parametrised for temperature and fatty acid composition. We remark that this study was performed in analytical scale vials with small oil columns, where we can assume that oxygen and oxidation products are homogeneously distributed. For larger containers such as jars, such assumption may not be made and diffusion of oxygen and oxidation products may need to be accounted for. We also note that the sunflower and rapeseed oils studied contained only low amounts of pro-oxidant transition metals. Next work should validate model predictions when higher concentrations of transition metals are present. In this work, the principle that a simpler kinetic network is a better network turned out to be valid. This principle will need to be validated again when the model is extended to, for example, polyunsaturated fatty acids.

## 4 Conclusions

The presented parametrised reaction network allows for quantitative *in silico* predictions of vegetable oil autoxidation under different environmental conditions, such as initial oxygen concentration, container dimension, and temperature during autoxidation. This parametrisation enables estimation of shelf-life for different packaging formats and conditions within supply chain and consumer use. The model can also handle variations in the fatty acid composition of common vegetable oils, and starting concentrations of hydroperoxides. These compositional factors can be obtained by straightforward chemical analyses methods. We consider the presented parametrised model pivotal for rationalising shelf-life predictions for vegetable oils. For oils comprising other polyunsaturated fatty acids, the reaction network can easily be used to determine their activation energies. This will enable parametrisation for a broader range of edible oils, we also envisage extension to applications to emulsified foods.

## CRediT authorship contribution statement

**Vincent J.P. Boerkamp:** Conceptualization, Investigation, Methodology, Validation, Visualisation, Writing – original draft. **Khoa A. Nguyen:** Conceptualization, Methodology, Software, Validation, Writing – review & editing. **Jean-Paul Vincken:** Writing – review & editing, Funding acquisition. **John P.M. van Duynhoven:** Conceptualization, Supervision, Writing – review & editing, Funding acquisition. **Marie Hennebelle:** Conceptualization, Supervision, Writing – review & editing.

## Declaration of competing interest

The authors declare the following financial interests/personal relationships which may be considered as potential competing interests: John P.M. van Duynhoven reports a relationship with the Unilever Foods Innovation Centre Wageningen that includes: employment.

## Data Availability

Source code and scripts have been deposited on Zenodo https://doi.org/10.5281/zenodo.15674464.

## Appendix A Supplementary data

MMC S1 Supplementary data supporting main findings, including experimental quantification of lipid oxidation products and tocopherols, figures, and tables.
