# Supplementary material for: Predictive mechanistic modelling of vegetable oil autoxidation using a parametrised kinetic network
Source: Curr Res Food Sci. 2025 Oct 30;11:101233. doi: 10.1016/j.crfs.2025.101233 (PMC12637084; doi:10.1016/j.crfs.2025.101233)
Supplement: MMC S1 — Supplementary data supporting main findings, including experimental quantification of lipid oxidation products and tocopherols, figures, and tables. [file mmc1.pdf]

Supporting information

Predictive mechanistic modelling of vegetable oil autoxidation using a parametrised kinetic network

Vincent J. P. Boerkamp,[a] Khoa A. Nguyen,[a] Jean-Paul Vincken,[a] John P. M. van Duynhoven,[b,c] and Marie Hennebelle[a]

[a] Vincent J. P. Boerkamp, Dr. Khoa A. Nguyen, Prof. Dr. Jean-Paul Vincken, Dr. Marie Hennebelle*
Laboratory of Food Chemistry, Wageningen University & Research, Wageningen, the Netherlands.
E-mail: vincent.boerkamp@wur.nl, jean-paul.vincken@wur.nl, marie.hennebelle@wur.nl

[b] Prof. Dr. John P. M. van Duynhoven
Laboratory of Biophysics, Wageningen University & Research, Wageningen, the Netherlands.
E-mail: john.vanduynhoven@wur.nl

[c] Prof. Dr. John P. M. van Duynhoven
Unilever Food Innovation Centre, Wageningen, the Netherlands.

S1. Experimental

S1.1. Lipid oxidation product quantification

The oxidised samples (50 mg for triolein, trilinolein, and trilinolenin, and 150 µL for all other samples) were dissolved in 5:1 $CDCl_3$:DMSO-d_6 to a total volume of 600 µL and transferred to 5 mm NMR tubes. Prior to sample preparation, the $CDCl_3$:DMSO-d_6 was dried over deuterated 4 Å molecular sieves to remove residual water. NMR spectra were recorded on a 600 MHz (14.1 T) Bruker Avance III NMR spectrometer (Bruker BioSpin, Switzerland) equipped with a cryo-probe or on a 700 MHz (16.4 T) Bruker Avance III HD NMR equipped with a room temperature probe operating at 295 K. All spectra were automatically phased, baseline corrected (third order polynomial), and integrated in MestReNova v14.1 (Mestrelab Research, S.L., Santiago de Compostela, Spain). TMS was used as spectral reference (δ_H 0 ppm).

Hydroperoxides and aldehydes were quantified by using single pulse and band selective ^{1}H NMR spectra, as previously described[1,2]. In contrast to our previous works, the C2 hydrogens (2.31 ppm) were used as the internal reference for quantification. This reduced the interference of C**H**(OOH)R_1R_2 signals upon oxidation that resonate between 4.7 and 3.7 ppm.

The non-oxidised TAG content (LH) was determined based on the full ^{1}H NMR spectra. Hereto, the number of non-oxidised double bonds was estimated by the allylic protons (2.02 ppm). The allylic protons were preferred over the bisallylic protons (2.8 ppm) as the allylic region was less crowded with oxidation products. Still, some overlapping signal was present for which a correction was required. For triolein, the allylic proton intensity was corrected for the LOOH formation, as two hydrogens of both *E*-HPOME (hydroperoxyoctadecenoate, 2.05 ppm) and *Z*-HPOME (2.098 ppm) remain with a chemical

shift in that region. The hydroxides, ketones, aldehydes, and epoxides were not interfering in this region. For trilinolein, the allylic proton intensity was corrected for two remaining hydrogens from *E*,*E*-HPODE (hydroperoxyoctadecadienoate). For trilinolenin, the allylic signal was corrected for overlap with the H8 of 12-HPOTrE (hydroperoxyoctadecatrienoate), the H8 of 16-HPOTrE, the H17 of 9-HPOTrE, the H17 of 13-HPOTrE, the H11 of diEP-16-HPOME (9,10-12,13-diepoxy-14*E*-16-hydroperoxyoctadecenoate), and the H14 of diEP-9-HPOME (11,12-15,16-diepoxy-10*E*-9-hydroperoxyoctadecenoate). Some unknown signals at δ_C/δ_H 48.0/2.09 ppm were also overlapping, however, as the structure was unknown (*i.e.*, the number of hydrogens), no correction was done for this structure. Finally, the concentration of non-oxidised LH was estimated by dividing the allylic proton integral (I_{LH}) by the concentration of non-oxidised fatty acid residues, which was estimated by the C2 proton (2.31 ppm) integral (I_{C2}).

$$c_{\mathrm{LH}}(\mathrm{mmol/kg\ TAG}) = \frac{I_{\mathrm{LH}}}{I_{\mathrm{C2}}} \cdot \frac{N_{\mathrm{C2}}}{N_{\mathrm{LH}}} \cdot \frac{10^6}{\mathrm{MW_{TG}}} - \mathrm{correction} \quad \text{(S1)}$$

where MW_{TG} is the molecular weight of the TAG. The C2 signal was not corrected for triolein, and trilinolein, whereas for trilinolenin it was corrected for two hydrogens of prostaglandin-like structures.

Epoxides, hydroxides, and ketones were quantified using ^{13}C band-selective 2D ^{1}H-^{13}C HSQC NMR spectroscopy based on our previous work[2,3]. The correction factor for hydroxides, and ketones was assumed to be equal to the factor for epoxides (1.16). The upfield triglyceride signal was referenced to δ_C 62.0 ppm, δ_H 4.13 ppm. The chemical shifts of the epoxides, hydroxides, and ketones were based on literature[2,4].

S1.2. Tocopherol quantification

Tocopherols were extracted as previously described[5]. In short, tocopherol was extracted by mixing 100 µL oil with 4 mL methanol. The mixture was shaken for 15 min and then centrifuged for 5 min at 4,690× g. Subsequently, the upper fraction (methanol) was collected and the same methanol extraction was repeated two more times on the oil fraction. The methanol fractions were combined and dried under nitrogen flow at 30 °C. The obtained extracts were stored at −20 °C until further analysis.

Tocopherols were separated by liquid chromatography according to Moltó-Puigmartí et al.[6]. Prior to analysis, the extracts were redissolved in 1 mL acetonitrile:methanol (80/20, v/v) by sonication for 10 min. Insoluble material was removed by centrifuging the mixtures for 5 min at 4,690x g. Of the supernatant, 1 µL was injected into a UPLC system (Thermo Scientific, San Jose, CA, USA) with an Acquity UPLC BEH C18 column (2.1 mm i.d. by 75 mm, 1.7 µm particle size) attached to an Acquity UPLC BEH C18 VanGuard Pre-column (2.1 mm i.d. by 5 mm, 1.7 µm particle size) (Waters, Milford, MA, USA) and isocratic elution with a binary eluent mix of acetonitrile:methanol (80/20, v/v). The flow rate was set to 0.6 mL/min, the column temperature to 30 °C, and the injection temperature to 10 °C. Tocopherols were detected using a photodiode array (PDA) detector at a wavelength of 292 nm and quantified based on calibration curves of pure standards.

S2. Supplementary figures

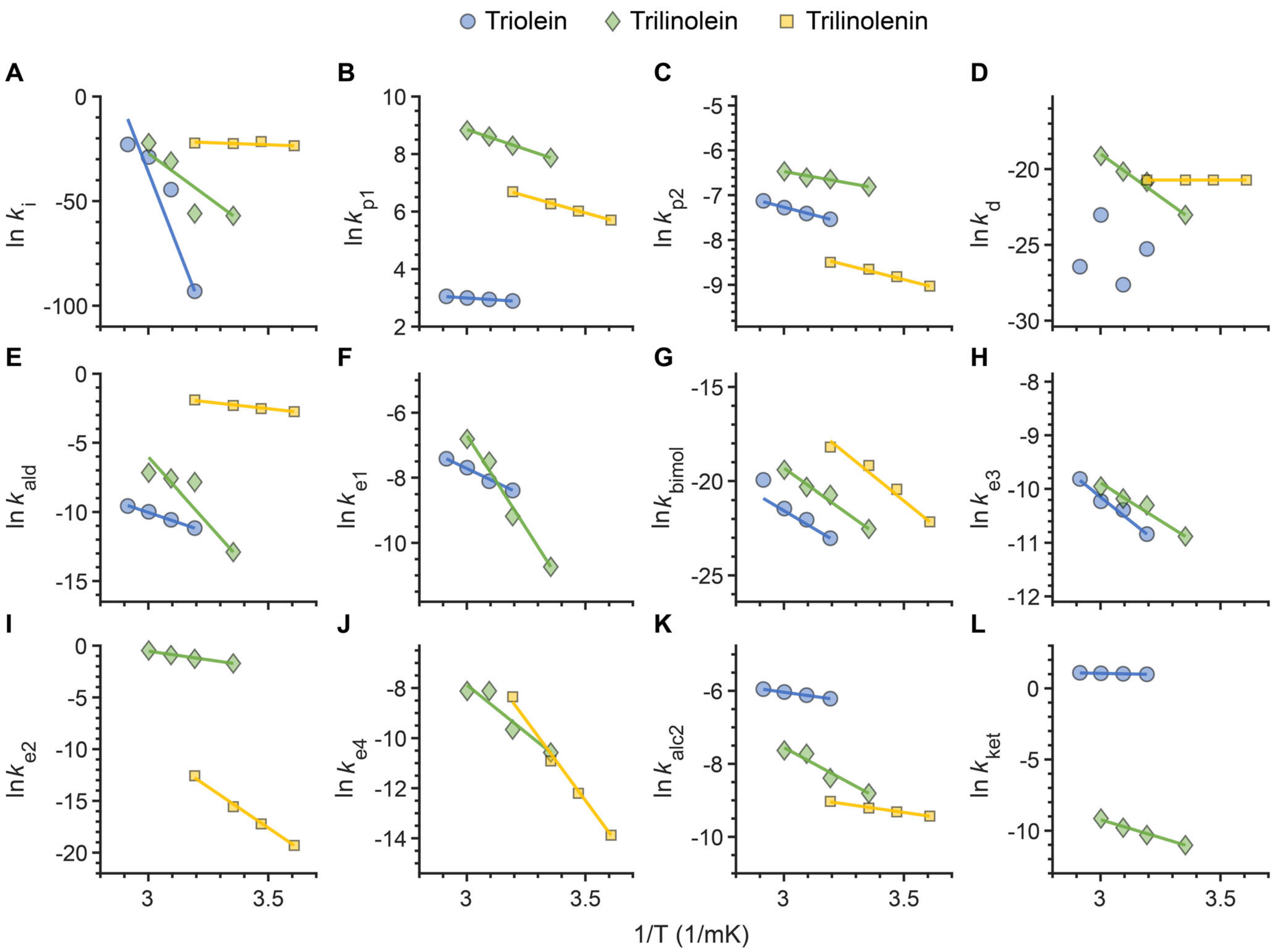

Figure S1. Arrhenius curves to estimate the activation energy of each reaction. The average error on the estimated rate constants was smaller than $10\,\%$. The activation energies were not estimated for k_{pro1}, k_{pro2}, and k_{loh1} as the model output was insensitive to these kinetic constants.

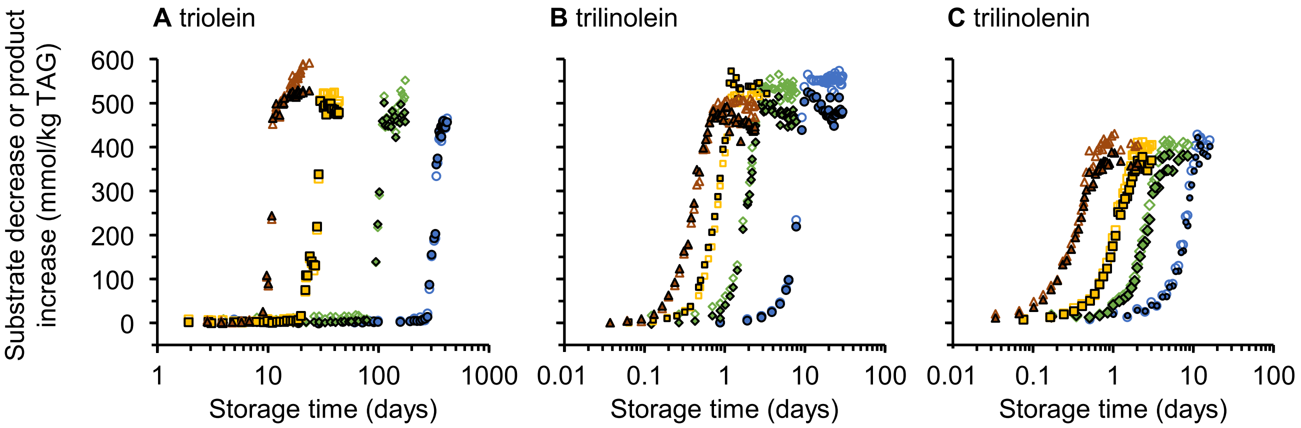

Figure S2. The total concentration of oxidation products (closed symbols) and the decrease in unoxidised substrate (open symbols) during the oxidation of triolein, trilinolein, and trilinolenin. From left to right in (A) 70, 60, 50, and $40\,^{\circ}\mathrm{C}$, (B) 60, 50, 40, $25\,^{\circ}\mathrm{C}$, and (C) 40, 25, 15, and $4\,^{\circ}\mathrm{C}$.

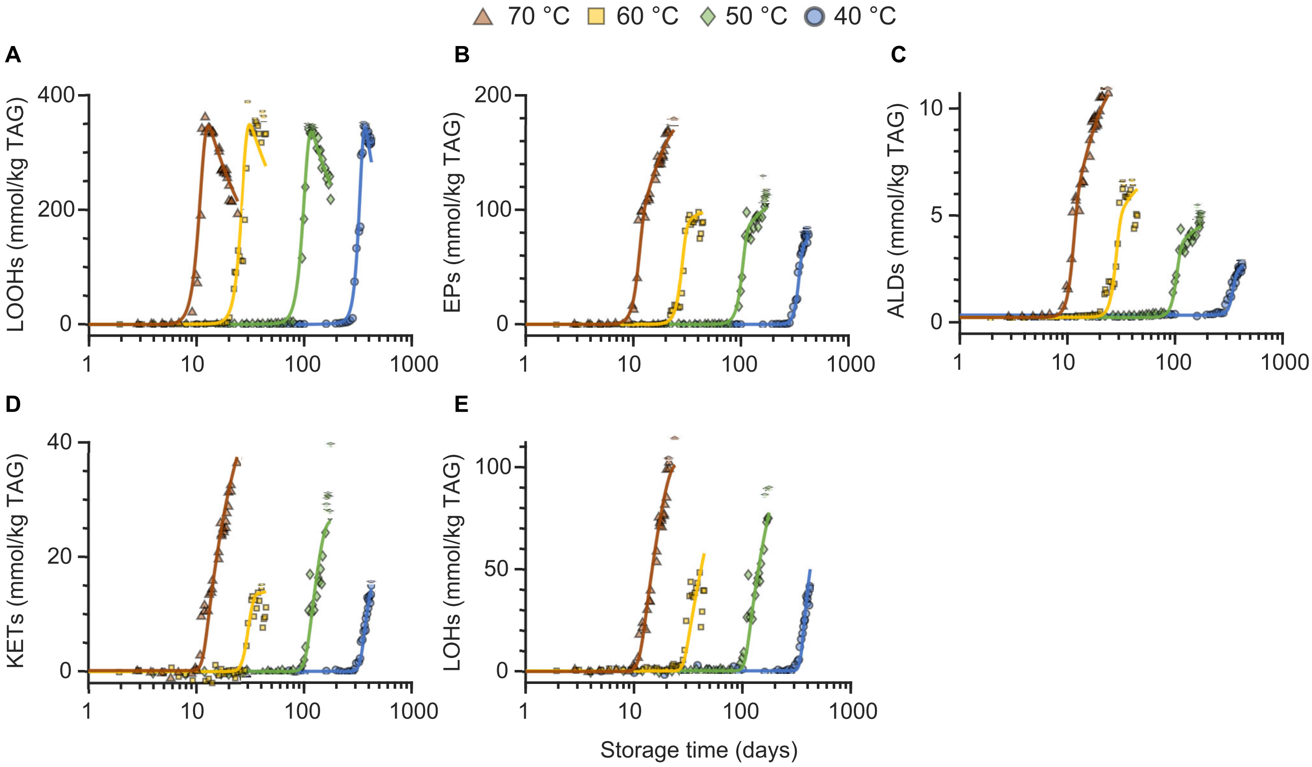

Figure S3. Estimating the rate constants of oxidation reactions in triolein at four temperatures by fitting the formation of lipid (A) hydroperoxides, (B) epoxides, (C) aldehydes, (D) ketones, and (E) hydroxides. The circles are experimental data and lines are outcomes of global fits.

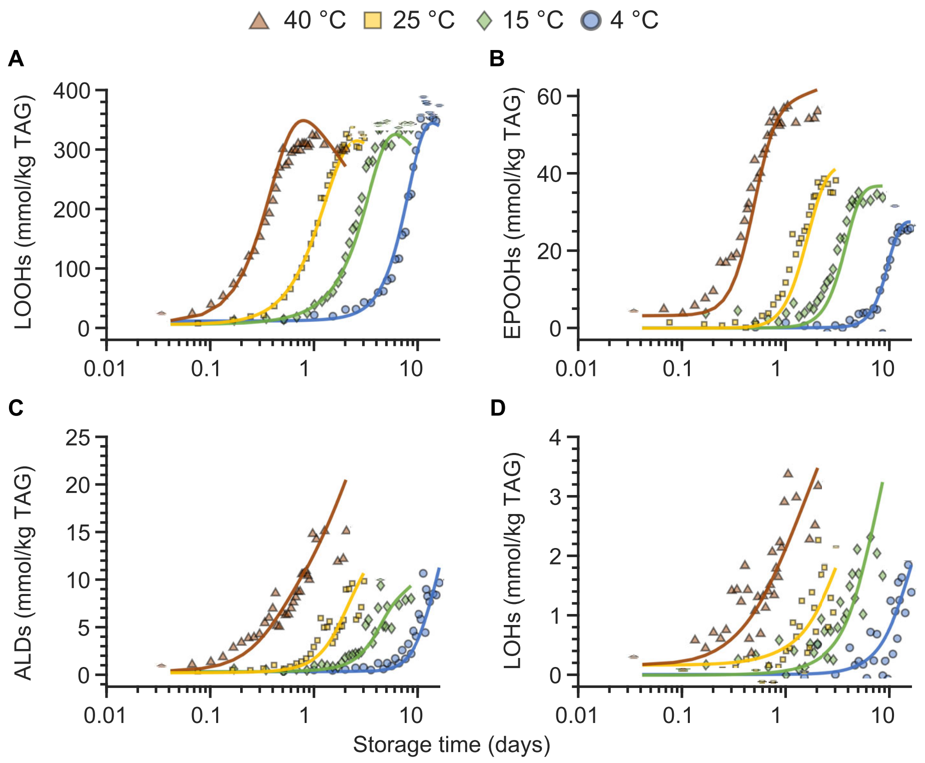

Figure S4. Estimating the rate constants of oxidation reactions in trilinolenin at four temperatures by fitting the formation of lipid (A) hydroperoxides, (B) epoxide-hydroperoxides, (C) aldehydes, and (D) hydroxides. The circles are experimental data and lines are outcomes of global fits.

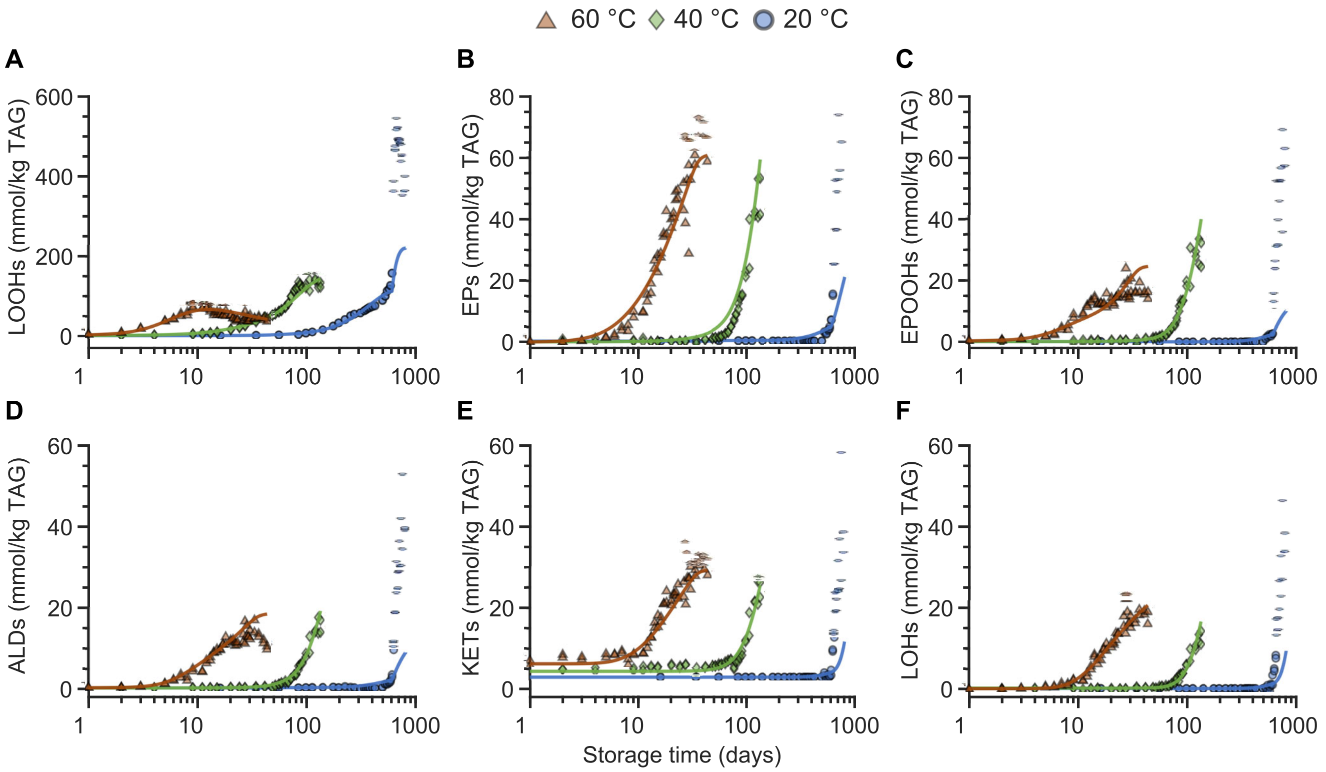

Figure S5. Modelling lipid oxidation of rapeseed oil with endogenous tocopherols as antioxidant without acceleration at $20\,^{\circ}\mathrm{C}$ to describe the formation of lipid (A) hydroperoxides, (B) epoxides, (C) epoxide-hydroperoxides, (D) aldehydes, (E) ketones, and (F) hydroxides. The circles are experimental data, and the lines are the model predictions. The oil ($1\,\mathrm{mL}$) was stored in the dark in closed vials ($20\,\mathrm{mL}$).

S3. Supplementary tables

Table S1. Kinetic rate constants for triolein. The relative standard error was determined to be approximately 5 % by Monte Carlo simulations. In our optimisation procedure, the starting values of the kinetic constants were first set to values in the range of $>10^{-13}$ (except for k_i). As the model outcome was insensitive to k_{pro2}, the same value was obtained at every temperature. k_{e2}, and k_{e4} were not estimated due to a too low EPOOH concentration.

Rate constant	Unit	Temperature (°C)			
		40	50	60	70
k_i	s^{-1}	$3.7{\cdot}10^{-41}$	$4.7{\cdot}10^{-20}$	$2.8{\cdot}10^{-13}$	$1.1{\cdot}10^{-10}$
k_{p1}	$m^3\ mol^{-1}\ s^{-1}$	$1.8{\cdot}10^{1}$	$1.9{\cdot}10^{1}$	$2.0{\cdot}10^{1}$	$2.1{\cdot}10^{1}$
k_{p2}	$m^3\ mol^{-1}\ s^{-1}$	$5.3{\cdot}10^{-4}$	$6.1{\cdot}10^{-4}$	$6.9{\cdot}10^{-4}$	$8.0{\cdot}10^{-4}$
k_d	s^{-1}	$1.1{\cdot}10^{-11}$	$1.0{\cdot}10^{-12}$	$1.0{\cdot}10^{-10}$	$3.3{\cdot}10^{-12}$
k_{bimol}	$m^3\ mol^{-1}\ s^{-1}$	$1.0{\cdot}10^{-10}$	$2.7{\cdot}10^{-10}$	$4.9{\cdot}10^{-10}$	$2.2{\cdot}10^{-9}$
k_{pro2}	$m^3\ mol^{-1}\ s^{-1}$	$5.0{\cdot}10^{-13}$	$5.0{\cdot}10^{-13}$	$5.0{\cdot}10^{-13}$	$5.0{\cdot}10^{-13}$
k_{e1}	$m^3\ mol^{-1}\ s^{-1}$	$2.3{\cdot}10^{-4}$	$3.0{\cdot}10^{-4}$	$4.6{\cdot}10^{-4}$	$6.0{\cdot}10^{-4}$
k_{e2}	$m^6\ mol^{-2}\ s^{-1}$	—	—	—	—
k_{e3}	$m^3\ mol^{-1}\ s^{-1}$	$2.0{\cdot}10^{-5}$	$3.1{\cdot}10^{-5}$	$3.6{\cdot}10^{-5}$	$5.5{\cdot}10^{-5}$
k_{e4}	$m^3\ mol^{-1}\ s^{-1}$	—	—	—	—
k_{ald}	$m^3\ mol^{-1}\ s^{-1}$	$1.4{\cdot}10^{-5}$	$2.6{\cdot}10^{-5}$	$4.6{\cdot}10^{-5}$	$7.0{\cdot}10^{-5}$
k_{loh1}	$m^3\ mol^{-1}\ s^{-1}$	$9.9{\cdot}10^{-10}$	$9.8{\cdot}10^{-10}$	$7.7{\cdot}10^{-10}$	$1.0{\cdot}10^{-10}$
k_{loh2}	$m^3\ mol^{-1}\ s^{-1}$	$2.0{\cdot}10^{-3}$	$2.2{\cdot}10^{-3}$	$2.4{\cdot}10^{-3}$	$2.6{\cdot}10^{-3}$
k_{ket}	$m^3\ mol^{-1}\ s^{-1}$	$2.7{\cdot}10^{0}$	$2.8{\cdot}10^{0}$	$2.9{\cdot}10^{0}$	$3.0{\cdot}10^{0}$

Table S2. Kinetic rate constants for trilinolein. The relative standard error was determined to be approximately 5 % by Monte Carlo simulations. In our optimisation procedure, the starting values of the kinetic constants were first set to values in the range of $>10^{-13}$ (except for k_i). As the model outcome was insensitive to k_{pro2}, the same value was obtained at every temperature.

Rate constant	Unit	Temperature (°C)			
		25	40	50	60
k_i	s^{-1}	$1.7{\cdot}10^{-25}$	$5.1{\cdot}10^{-25}$	$3.3{\cdot}10^{-14}$	$2.2{\cdot}10^{-10}$
k_{p1}	$m^3\ mol^{-1}\ s^{-1}$	$2.6{\cdot}10^{3}$	$4.0{\cdot}10^{3}$	$5.5{\cdot}10^{3}$	$6.8{\cdot}10^{3}$
k_{p2}	$m^3\ mol^{-1}\ s^{-1}$	$1.1{\cdot}10^{-3}$	$1.3{\cdot}10^{-3}$	$1.4{\cdot}10^{-3}$	$1.6{\cdot}10^{-3}$
k_d	s^{-1}	$1.0{\cdot}10^{-10}$	$8.9{\cdot}10^{-10}$	$1.8{\cdot}10^{-9}$	$4.9{\cdot}10^{-9}$
k_{bimol}	$m^3\ mol^{-1}\ s^{-1}$	$1.6{\cdot}10^{-10}$	$9.9{\cdot}10^{-10}$	$1.5{\cdot}10^{-9}$	$3.7{\cdot}10^{-9}$
k_{pro2}	$m^3\ mol^{-1}\ s^{-1}$	$5.0{\cdot}10^{-13}$	$5.0{\cdot}10^{-13}$	$5.0{\cdot}10^{-13}$	$5.0{\cdot}10^{-13}$
k_{e1}	$m^3\ mol^{-1}\ s^{-1}$	$2.2{\cdot}10^{-5}$	$1.0{\cdot}10^{-4}$	$5.5{\cdot}10^{-4}$	$1.1{\cdot}10^{-3}$
k_{e2}	$m^6\ mol^{-2}\ s^{-1}$	$1.8{\cdot}10^{-1}$	$2.8{\cdot}10^{-1}$	$4.0{\cdot}10^{-1}$	$6.3{\cdot}10^{-1}$
k_{e3}	$m^3\ mol^{-1}\ s^{-1}$	$1.9{\cdot}10^{-5}$	$3.4{\cdot}10^{-5}$	$3.8{\cdot}10^{-5}$	$4.8{\cdot}10^{-5}$
k_{e4}	$m^3\ mol^{-1}\ s^{-1}$	$2.6{\cdot}10^{-5}$	$6.4{\cdot}10^{-5}$	$3.0{\cdot}10^{-4}$	$3.0{\cdot}10^{-4}$
k_{ald}	$m^3\ mol^{-1}\ s^{-1}$	$2.5{\cdot}10^{-6}$	$3.9{\cdot}10^{-4}$	$5.1{\cdot}10^{-4}$	$7.7{\cdot}10^{-4}$
k_{loh1}	$m^3\ mol^{-1}\ s^{-1}$	$1.0{\cdot}10^{-9}$	$1.0{\cdot}10^{-9}$	$1.0{\cdot}10^{-9}$	$1.0{\cdot}10^{-9}$
k_{loh2}	$m^3\ mol^{-1}\ s^{-1}$	$1.5{\cdot}10^{-4}$	$2.3{\cdot}10^{-4}$	$4.4{\cdot}10^{-4}$	$4.8{\cdot}10^{-4}$
k_{ket}	$m^3\ mol^{-1}\ s^{-1}$	$1.6{\cdot}10^{-5}$	$3.3{\cdot}10^{-5}$	$5.6{\cdot}10^{-5}$	$1.1{\cdot}10^{-4}$

Table S3. Kinetic rate constants for trilinolenin. The relative standard error was determined to be approximately $5\,\%$ by Monte Carlo simulations. In our optimisation procedure, the starting values of the kinetic constants were first set to values in the range of $>10^{-13}$ (except for k_i). As the model outcome was insensitive to k_pro2, the same value was obtained at every temperature. k_e1, k_e3, and k_ket were not estimated due to a too low product concentration.

Rate constant	Unit	Temperature (°C)			
		4	15	25	40
k_i	s^{-1}	$6.1{\cdot}10^{-11}$	$4.4{\cdot}10^{-10}$	$1.7{\cdot}10^{-10}$	$2.2{\cdot}10^{-10}$
k_p1	$m^3\ mol^{-1}\ s^{-1}$	$3.0{\cdot}10^{2}$	$4.1{\cdot}10^{2}$	$5.3{\cdot}10^{2}$	$8.0{\cdot}10^{2}$
k_p2	$m^3\ mol^{-1}\ s^{-1}$	$1.2{\cdot}10^{-4}$	$1.5{\cdot}10^{-4}$	$1.7{\cdot}10^{-4}$	$2.0{\cdot}10^{-4}$
k_d	s^{-1}	$1.0{\cdot}10^{-9}$	$1.0{\cdot}10^{-9}$	$1.0{\cdot}10^{-9}$	$1.0{\cdot}10^{-9}$
k_bimol	$m^3\ mol^{-1}\ s^{-1}$	$2.4{\cdot}10^{-10}$	$1.3{\cdot}10^{-9}$	$4.7{\cdot}10^{-9}$	$1.3{\cdot}10^{-8}$
k_pro2	$m^3\ mol^{-1}\ s^{-1}$	$5.0{\cdot}10^{-13}$	$5.0{\cdot}10^{-13}$	$5.0{\cdot}10^{-13}$	$5.0{\cdot}10^{-13}$
k_e1	$m^3\ mol^{-1}\ s^{-1}$	—	—	—	—
k_e2	$m^6\ mol^{-2}\ s^{-1}$	$4.1{\cdot}10^{-9}$	$3.3{\cdot}10^{-8}$	$1.7{\cdot}10^{-7}$	$3.5{\cdot}10^{-6}$
k_e3	$m^3\ mol^{-1}\ s^{-1}$	—	—	—	—
k_e4	$m^3\ mol^{-1}\ s^{-1}$	$9.4{\cdot}10^{-7}$	$5.0{\cdot}10^{-6}$	$1.8{\cdot}10^{-5}$	$2.4{\cdot}10^{-4}$
k_ald	$m^3\ mol^{-1}\ s^{-1}$	$5.2{\cdot}10^{-1}$	$2.8{\cdot}10^{-1}$	$1.8{\cdot}10^{-1}$	$1.1{\cdot}10^{-1}$
k_loh1	$m^3\ mol^{-1}\ s^{-1}$	$1.0{\cdot}10^{-9}$	$1.0{\cdot}10^{-9}$	$1.0{\cdot}10^{-9}$	$1.0{\cdot}10^{-9}$
k_loh2	$m^3\ mol^{-1}\ s^{-1}$	$3.4{\cdot}10^{-4}$	$1.7{\cdot}10^{-4}$	$1.0{\cdot}10^{-4}$	$4.3{\cdot}10^{-5}$
k_ket	$m^3\ mol^{-1}\ s^{-1}$	—	—	—	—

Table S4. The kinetic rate constants (k) were estimated at $20\,°C$ by using the Arrhenius equation. The multiplication factors f were estimated using $k_{C_\mathrm{LOOH,critical}} = f \times k$ by the model fitting to a second acceleration phase in lipid oxidation reactions when the LOOH concentration reached a certain critical LOOH concentration ($C_\mathrm{LOOH,critical}$). In our optimisation procedure, $C_\mathrm{LOOH,critical}$ was also estimated to be $113\,\mathrm{mmol/kg}$ TAG.

Rate constant	Triolein		Trilinolein		Trilinolenin	
	k	f	k	f	k	f
k_i	$4.5 \cdot 10^{-69}$	1	$4.7 \cdot 10^{-13}$	1	$1.4 \cdot 10^{-10}$	1
k_p1	$1.6 \cdot 10^{1}$	17	$2.2 \cdot 10^{3}$	8	$4.7 \cdot 10^{2}$	14
k_p2	$3.9 \cdot 10^{-4}$	151	$1.0 \cdot 10^{-3}$	10	$1.6 \cdot 10^{-4}$	12
k_d	$1.0 \cdot 10^{-12}$	1	$5.2 \cdot 10^{-11}$	1	$1.0 \cdot 10^{-9}$	1
k_bimol	$1.1 \cdot 10^{-11}$	21	$9.8 \cdot 10^{-11}$	21	$1.8 \cdot 10^{-9}$	21
k_pro2	$5.0 \cdot 10^{-13}$	1	$5.0 \cdot 10^{-13}$	1	$5.0 \cdot 10^{-13}$	1
k_e1	$1.1 \cdot 10^{-4}$	100	$1.1 \cdot 10^{-5}$	200	—	—
k_e2	—	—	$1.5 \cdot 10^{-1}$	1	$9.2 \cdot 10^{-8}$	1
k_e3	$9.0 \cdot 10^{-6}$	1	$1.6 \cdot 10^{-5}$	1	—	—
k_e4	—	—	$1.7 \cdot 10^{-5}$	930000	$1.2 \cdot 10^{-5}$	21
k_ald	$3.9 \cdot 10^{-6}$	50	$8.2 \cdot 10^{-7}$	400	$9.4 \cdot 10^{-2}$	177
k_loh1	$1.0 \cdot 10^{-9}$	1	$1.0 \cdot 10^{-9}$	1	$1.0 \cdot 10^{-9}$	1
k_loh2	$1.6 \cdot 10^{-3}$	1	$1.2 \cdot 10^{-4}$	1	$9.6 \cdot 10^{-5}$	1
k_ket	$0.2 \cdot 10^{1}$	1	$1.2 \cdot 10^{-5}$	1	—	—

Table S5. The kinetic rate constants (k) of reactions with tocopherols in rapeseed oil as estimated by the model fitting to early short-term formation of LOOHs (Figure 4A). In our optimisation procedure, the starting values of the kinetic constants of reactions with tocopherols were first set to values in the range of $5 \cdot 10^{-13}$. As the model outcome was insensitive to k_{AH3} and k_{t4}, the same value was obtained at every temperature. This insensitivity was in line with the diffusion-controlled rate for addition of oxygen to $L^{\bullet}$. This reaction will therefore outcompete the hydrogen donation by an antioxidant[7].

Rate constant	Temperature (°C)	Triolein	Trilinolein	Trilinolenin
k_{AH1}	20	$8.0 \cdot 10^{-2}$	$1.0 \cdot 10^{-1}$	$1.5 \cdot 10^{-1}$
	40	$1.6 \cdot 10^{-1}$	$2.0 \cdot 10^{-1}$	$3.0 \cdot 10^{-1}$
	60	$3.0 \cdot 10^{-1}$	$4.0 \cdot 10^{-1}$	$6.0 \cdot 10^{-1}$
k_{AH2}	20	$8.0 \cdot 10^{-2}$	$2.7 \cdot 10^{2}$	$4.2 \cdot 10^{2}$
	40	$1.6 \cdot 10^{-1}$	$5.4 \cdot 10^{2}$	$8.4 \cdot 10^{2}$
	60	$3.0 \cdot 10^{-1}$	$1.1 \cdot 10^{3}$	$1.7 \cdot 10^{3}$
k_{AH3}	20	$5.0 \cdot 10^{-13}$	$5.0 \cdot 10^{-13}$	$5.0 \cdot 10^{-13}$
	40	$5.0 \cdot 10^{-13}$	$5.0 \cdot 10^{-13}$	$5.0 \cdot 10^{-13}$
	60	$5.0 \cdot 10^{-13}$	$5.0 \cdot 10^{-13}$	$5.0 \cdot 10^{-13}$
k_{t4}	20	$5.0 \cdot 10^{-13}$	$5.0 \cdot 10^{-13}$	$5.0 \cdot 10^{-13}$
	40	$5.0 \cdot 10^{-13}$	$5.0 \cdot 10^{-13}$	$5.0 \cdot 10^{-13}$
	60	$5.0 \cdot 10^{-13}$	$5.0 \cdot 10^{-13}$	$5.0 \cdot 10^{-13}$
